# Multidisciplinary team directed analysis of whole genome sequencing reveals pathogenic non-coding variants in molecularly undiagnosed inherited retinal dystrophies

**DOI:** 10.1093/hmg/ddac227

**Published:** 2022-09-09

**Authors:** Malena Daich Varela, James Bellingham, Fabiana Motta, Neringa Jurkute, Jamie M Ellingford, Mathieu Quinodoz, Kathryn Oprych, Michael Niblock, Lucas Janeschitz-Kriegl, Karolina Kaminska, Francesca Cancellieri, Hendrik P N Scholl, Eva Lenassi, Elena Schiff, Hannah Knight, Graeme Black, Carlo Rivolta, Michael E Cheetham, Michel Michaelides, Omar A Mahroo, Anthony T Moore, Andrew R Webster, Gavin Arno

**Affiliations:** UCL Institute of Ophthalmology, London EC1V 9EL, UK; Moorfields Eye Hospital, London EC1V 2PD, UK; UCL Institute of Ophthalmology, London EC1V 9EL, UK; UCL Institute of Ophthalmology, London EC1V 9EL, UK; Department of Ophthalmology, Universidade Federal de Sao Paulo, Sao Paulo 04021001, Brazil; UCL Institute of Ophthalmology, London EC1V 9EL, UK; Moorfields Eye Hospital, London EC1V 2PD, UK; North West Genomic Laboratory Hub, Manchester Centre for Genomic Medicine, Manchester University Hospitals NHS Foundation Trust, St Mary’s Hospital, Manchester M13 9WL, UK; Division of Evolution and Genomic Sciences, Neuroscience and Mental Health Domain, School of Biological Sciences, Faculty of Biology, Medicine and Health, University of Manchester, Manchester M13 9PL, UK; Institute of Molecular and Clinical Ophthalmology Basel, Basel 4031, Switzerland; Department of Ophthalmology, University of Basel, Basel 4031, Switzerland; Department of Genetics and Genome Biology, University of Leicester, Leicester LE1 7RH, UK; UCL Institute of Ophthalmology, London EC1V 9EL, UK; UCL Institute of Ophthalmology, London EC1V 9EL, UK; Institute of Molecular and Clinical Ophthalmology Basel, Basel 4031, Switzerland; Department of Ophthalmology, University of Basel, Basel 4031, Switzerland; Institute of Molecular and Clinical Ophthalmology Basel, Basel 4031, Switzerland; Department of Ophthalmology, University of Basel, Basel 4031, Switzerland; Institute of Molecular and Clinical Ophthalmology Basel, Basel 4031, Switzerland; Department of Ophthalmology, University of Basel, Basel 4031, Switzerland; Institute of Molecular and Clinical Ophthalmology Basel, Basel 4031, Switzerland; Department of Ophthalmology, University of Basel, Basel 4031, Switzerland; North West Genomic Laboratory Hub, Manchester Centre for Genomic Medicine, Manchester University Hospitals NHS Foundation Trust, St Mary’s Hospital, Manchester M13 9WL, UK; Division of Evolution and Genomic Sciences, Neuroscience and Mental Health Domain, School of Biological Sciences, Faculty of Biology, Medicine and Health, University of Manchester, Manchester M13 9PL, UK; Moorfields Eye Hospital, London EC1V 2PD, UK; Moorfields Eye Hospital, London EC1V 2PD, UK; North West Genomic Laboratory Hub, Manchester Centre for Genomic Medicine, Manchester University Hospitals NHS Foundation Trust, St Mary’s Hospital, Manchester M13 9WL, UK; Division of Evolution and Genomic Sciences, Neuroscience and Mental Health Domain, School of Biological Sciences, Faculty of Biology, Medicine and Health, University of Manchester, Manchester M13 9PL, UK; Institute of Molecular and Clinical Ophthalmology Basel, Basel 4031, Switzerland; Department of Ophthalmology, University of Basel, Basel 4031, Switzerland; Department of Genetics and Genome Biology, University of Leicester, Leicester LE1 7RH, UK; UCL Institute of Ophthalmology, London EC1V 9EL, UK; UCL Institute of Ophthalmology, London EC1V 9EL, UK; Moorfields Eye Hospital, London EC1V 2PD, UK; UCL Institute of Ophthalmology, London EC1V 9EL, UK; Moorfields Eye Hospital, London EC1V 2PD, UK; UCL Institute of Ophthalmology, London EC1V 9EL, UK; Moorfields Eye Hospital, London EC1V 2PD, UK; University of California, San Francisco, CA 94607, USA; UCL Institute of Ophthalmology, London EC1V 9EL, UK; Moorfields Eye Hospital, London EC1V 2PD, UK; UCL Institute of Ophthalmology, London EC1V 9EL, UK; Moorfields Eye Hospital, London EC1V 2PD, UK; North Thames Genomic Laboratory Hub, Great Ormond Street Hospital For Children, London WC1N 3JH, UK

## Abstract

The purpose of this paper is to identify likely pathogenic non-coding variants in inherited retinal dystrophy (IRD) genes, using genome sequencing (GS). Patients with IRD were recruited to the study and underwent comprehensive ophthalmological evaluation and GS. The results of GS were investigated through virtual gene panel analysis, and plausible pathogenic variants and clinical phenotype evaluated by the multidisciplinary team (MDT) discussion. For unsolved patients in whom a specific gene was suspected to harbor a missed pathogenic variant, targeted re-analysis of non-coding regions was performed on GS data. Candidate variants were functionally tested by messenger RNA analysis, minigene or luciferase reporter assays. Previously unreported, likely pathogenic, non-coding variants in 7 genes (*PRPF31, NDP, IFT140*, *CRB1*, *USH2A*, *BBS10* and *GUCY2D*), were identified in 11 patients. These were shown to lead to mis-splicing (*PRPF31*, *IFT140*, *CRB1* and *USH2A*) or altered transcription levels (*BBS10* and *GUCY2D*). MDT-led, phenotype-driven, non-coding variant re-analysis of GS is effective in identifying the missing causative alleles.

## Introduction

Inherited retinal dystrophy (IRD) is a heterogeneous group of rare diseases that result in visual impairment caused by retinal dysfunction (1). To date, over 300 genes and loci have been associated with IRD (https://sph.uth.edu/retnet/), with a carrier frequency estimated to be up to approximately 1 in 2.5 individuals (2), and a prevalence of around 1 in 2000 people (3). IRD can be classified in many ways by taking into account the inheritance pattern, age at onset, rate of progression, main retinal cell type affected (rods, cones, retinal pigment epithelium, retinal ganglion cells or choroid), and extra-ocular features (4).

Genetic diagnosis of IRD is essential for effective clinical management, and more relevant now with the approval of the first gene therapy for *RPE65-*retinal dystrophy (Luxturna), and with many other clinical trials for these disorders in progress and development (5). Although some of the approaches such as optogenetics, retinal cell transplantation and artificial retinal prostheses) are gene agnostic (6–8), many of the current efforts focus on gene and RNA supplementation and editing, making genetic diagnosis a key inclusion criterion (9–12).

**Table 1:** Genetic and analysis details of our cohort of patients

ID	Gene	GRCh38 coordinates	Variant	gnomAD	*In silico* predictions	Experiment	Result
1	*PRPF31*	Chr19:54129939C > G	c.1374 + 569C > G Het	Absent	New splice donor site (nnsplice 0.00 > 0.61, SpliceAI donor gain score 0.74–3 bp)	Direct mRNA	88-bp pseudoexon insertion, p.(Gly459Serfs^*^46)
2	*PRPF31*	Chr19:54115798G > A	c.-9 + 1G > A Het	Absent	Abolish splice donor site (nnsplice 0.99 > 0.00, SpliceAI donor loss 0.99–1 bp; donor gain 0.66–44 bp)	N/A	N/A
3	*IFT140*	Chr16:1526044G > A Chr16:1526614_1526615del	c.2611C > T p.(Arg871Cys) c.2577 + 4_2577 + 5del	0.00001580 Absent	Pathogenic x2, VUS x1 (ClinVar ID 987304) Abolish splice donor site (nnsplice 1.00 > 0.00, Splice AI donor loss 0.71–5 bp; donor gain 0.1–16 bp )	- Direct mRNA	- Two cryptic splice transcripts: Exon 20 truncation p.(Val822_Leu859del) Exon 20 skipping p.(Ser800Argfs^*^16)
4	*NDP*	ChrX: 43958715C > T	c.-70G > A hemi	Absent	SpliceAI donor gain (score 0.40–3 bp)	Luciferase assay	Negative
5	*CRB1*	Chr1:197427615C > T Chr1:197440963C > G	c.2290C > T p.(Arg764Cys) c.3879-1203C > G	Absent Absent	N/A New splice donor site (+1 position C > G, nnsplice 0.00 > 0.96, SpliceAI donor gain 0.29–1 bp )	- Minigene analysis	- 156-bp cryptic exon inclusion, p.(Trp1293_Cys1294insPhe^*^)
6	*CRB1*	Chr1:197429619G > A Chr1:197440963C > G	c.2842 + 5G > A c.3879-1203C > G	0.00003188 Absent	Likely pathogenic (ClinVar ID 438078) New splice donor site (+1 position C > G, nnsplice 0.00 > 0.96, Splice AI donor gain 0.29–1 bp)	- Minigene analysis	- 156-bp cryptic exon inclusion, p.(Trp1293_Cys1294insPhe^*^)
7	*USH2A*	Chr1:216325412 T > G Chr1:216088638 T > C	c.1036A > C p.(Asn346His) c.4885 + 375A > G	0.00006729 Absent	Pathogenic (ClinVar ID 48347) Strengthen cryptic splice donor site (+5 position A > G, nnsplice 0.76 > 0.99, SpliceAI donor gain 0.67–5 bp)	- Direct mRNA assay (nasal epithelial)	- 130-bp pseudoexon insertion, p.(Gly1629Valfs^*^52)
8	*BBS10*	Chr12:76345866_76345867del Chr12:76348438dup	c.2119_2120del p.(Val707^*^) c.-80dup	0.00006 Absent	Pathogenic (Clinvar ID 406221) Located in core promoter region	- Luciferase assay	- 70% reduced expression
9	*GUCY2D*	Chr17:8015395C > A Chr17:8002596 T > C	c.2837C > A, p.(Ala946Glu) c.-148 T > C	Absent Absent	Uncertain significance (ClinVar ID 848290) Located in CRX binding site	- Luciferase assay	- 30% reduced expression
10	*CRB1*	Chr1:197427726A > T Chr1:197440963C > G	c.2401A > T p.(Lys801^*^) c.3879-1203C > G	0.0001183 0.00001972	Pathogenic (ClinVar ID 5736) New splice donor site (+1 position C > G, nnsplice 0.00 > 0.96, SpliceAI donor gain 0.29–1 bp )	- Minigene analysis	- 156-bp cryptic exon inclusion, p.(Cys1294Phefs^*^2)
11	*GUCY2D*	Chr17:8015846G > A Chr17:8002596 T > C	c.3043 + 5G > A GUCY2D c.-148 T > C	Absent Absent	Likely pathogenic (ClinVar ID1184616) Located in CRX binding site	- Luciferase assay	- 30% reduced expression

**Table 2:** Clinical details of our cohort of patients

ID	Phenotype	Sex—race	Symptoms and age of onset (years)	BCVA logMAR (age)	Visual field (age)	Fundus features	Macular OCT features	Family history
1	RCD	M	Nyctalopia (20)	0.2 OD & 0 OS (22)	~ 25° (23)	Pigmented bone spicules, RPE mottling, vessel thinning	Subfoveal island of outer segments, oedema	Yes, AD non-penetrant
2	RCD	M – Asian Indian	-	1.5 OD & 1.3 OS (23)	~ 10° (23)	Pigmented bone spicules, white spots, vessel thinning	Thin, overall loss of EZ line, few cysts	AD
3	CRD	M—White	Decreased acuity (24)	HM OD & 0.25 OS (25)	-	RPE atrophy and pigment clumping on posterior pole	Thin, loss of EZ line	No
4	Bilateral retinal folds/FEVR	M—White	Nystagmus and decreased acuity (birth)	1.6 OD and NLP OS (6)	-	Bilateral retinal folds	Thin retina, schisis	Yes (maternal grandfather)
5	EOSRD	M—White	Constricted visual field (10)	LP OD and OS (25)	~ 10° (26)	Diffuse pigment; peripheral telangiectasia, retinal exudate and tractional retinal detachment in OD	Right macular schisis, atrophy and loss of EZ line OU, blurred layers	Yes (brother and sister—no consanguinity)
6	EOSRD	M	Constricted visual field (5)	LP OD & OS (23)	-	Diffuse widespread pigment in bone spicules and nummules, peripheral atrophy	Thin, loss of EZ line, blurred layers	No
7	Usher syndrome type II	F—White	Nyctalopia (14) Hearing loss since small child (aids)	0.2 OD and 0.2 OS (27)	~ 25° (23)	Pigmented bone spicules, vessel thinning, pale optic disk	Subfoveal island of outer segments, oedema, right epiretinal membrane	No
8	CRD	F—White	Photoaversion (28)	1 OD and 0.7 OS (69)	-	RPE atrophy on posterior pole and peripheral patches	Subfoveal island of outer segments	No
9	EOSRD	M—White	Nystagmus and poor VA (birth)	1.8 OD and 1.5 OS (29)	-	Featureless fundus and macular atrophy	Disrupted subfoveal EZ line	Yes (sister)
10	EOSRD	F – White	Nyctalopia, constricted visual field	1 OD, 1.1 OS (16)	20° (16)	Para-arteriolar preservation of the RPE (FAF), bone spicules, attenuated vessels, generalized atrophy	Blurred inner retinal layers and retinal thickening, loss of outer retinal layers	No

RCD: rod-cone dystrophy; CRD: cone-rod dystrophy; FEVR: familial exudative vitreoretinopathy; EOSRD: early onset severe retinal dystrophy; F: feminine; M: masculine; BCVA: best corrected visual acuity; OD: right eye; OS: left eye; HM: hand movements; LP: light perception; NLP: no light perception; RPE: retinal pigment epithelium; FAF: fundus autofluorescence; OCT: optical coherence tomography; EZ: ellipsoid zone; AD: autosomal dominant.

Genetic testing in the ophthalmic genetics’ clinic has evolved dramatically over the last decades, as technologies have become more accessible and inexpensive. Single-candidate gene approach by Sanger sequencing, next generation sequencing (NGS)-based panels, exome sequencing (ES) and genome sequencing (GS) are currently employed by clinical diagnostic, commercial and research laboratories worldwide (13). GS represents the most comprehensive test, with a significant improvement in diagnostic yield compared with NGS panels (14) and ES (15). Yet, the pathogenic  variant detection rate in IRD ranges between 50 and 85% depending on patient cohorts (15–18). The missing genetic etiology has been attributed to undiscovered genes, novel damaging variants, copy number variants (CNV), synonymous changes that affect transcript processing and changes in non-coding regions (introns and regulatory) that are particularly difficult to interpret and functionally assay, especially for retina-specific genes (15,19–21). However, for molecular geneticists to truly capitalize on this technology, an improved interpretation and understanding of how or which non-protein-altering variants cause disease is required.

Here we present findings from large-scale GS studies in the UK: the National Institute for Health Research Rare Disease (NIHR-RD) and 100 000 Genomes Project (100KGP). We demonstrate that targeted investigations of non-coding variants in a case-led manner is effective for identification of missing variants in IRD.

## Results

Patient information including clinical findings and genetic details are summarized in Tables 1 and 2. Candidate gene non-coding variant analysis, following negative clinical testing and multidisciplinary team (MDT) discussion highlighting specific candidate genes in each case, led us to identify two patients with a candidate variant in *PRPF31*, one with a variant in *NDP*, two with a recurrent variant in *CRB1*, one variant in *USH2A*, 1 in *IFT140*, one in *BBS10* and one in *GUCY2D*.

**Figure 1:**
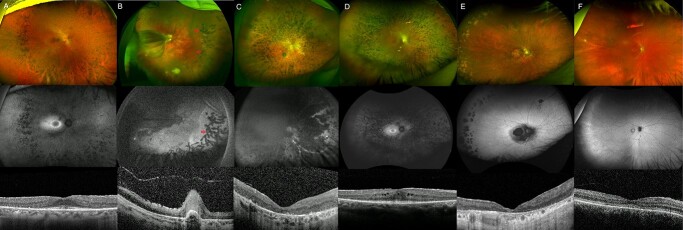
Retinal imaging from individuals with IRD within the analyzed cohort. (**A**) Ultrawide-field (UWF) colour fundus image showing pigmented bone spicules-like lesions and vessel thinning in patient 1, with *PRPF31*-associated RCD. Fundus autofluorescence (FAF) imaging is positive for a macular hyperautofluorescent ring, characteristic of rod-cone dystrophies (RCD), and generalized decreased hypoAF. Macular optical coherence tomography (OCT) shows a subfoveal island of outer layers. (**B**) UWF colour and FAF images from patient 4, a child with Norrie disease and bilateral retinal folds. Prophylactic bilateral pan-retinal photocoagulation spots are also visible, marked with red arrows. Macular OCT shows a retinal fold and poor retinal architecture. (**C**) UWF fundus and OCT imaging from patient 6, who has *CRB1*-early onset severe retinal dystrophy. Retinal images are positive for nummular, dense, deep pigment deposition, preserved autofluorescence adjacent to few peripheral retinal arterioles and a poorly laminated retina. (**D**) UWF colour and FAF imaging of patient 7, showing typical RCD features associated with *USH2A* retinopathy. We can see dense pigment deposits in the mid-periphery, a perifoveal hyperautofluorescent ring demarcating the area of functioning retina, and decreased peripheral AF. Macular OCT shows a subfoveal island of outer segments and cystoid macular oedema. (**E**) UWF colour, FAF and OCT imaging from patient 8, with *BBS10*-associated retinopathy. Retinal appearance shows a CRD pattern, with demarcated posterior pole and peripheral areas of retinal pigment epithelium atrophy. Macular OCT appears well correlated, with generalized loss of the outer layers. (**F**) UWF colour, FAF and OCT images of patient 9, diagnosed with *GUCY2D*-related EOSRD. Of note is the featureless fundus, mild vessel narrowing, macular atrophy, and disrupted ellipsoid zone centrally.

Patient 1 (GC 763) is a 34-year-old male with typical fundus features of rod-cone dystrophy (RCD), no extra-ocular manifestations suggesting syndromic disease and a family history indicative of autosomal dominant RCD (adRCD, Fig. 1A), with incomplete penetrance owing to the observed unaffected obligate carrier mother. *PRPF31*, a common cause of adRCD showing incomplete penetrance, was screened by direct Sanger sequencing and followed by NGS panel genetic testing for all known autosomal dominant RCD genes, which were negative. Subsequently, he was recruited for GS through the 100KGP with his unaffected parents.

**Figure 2:**
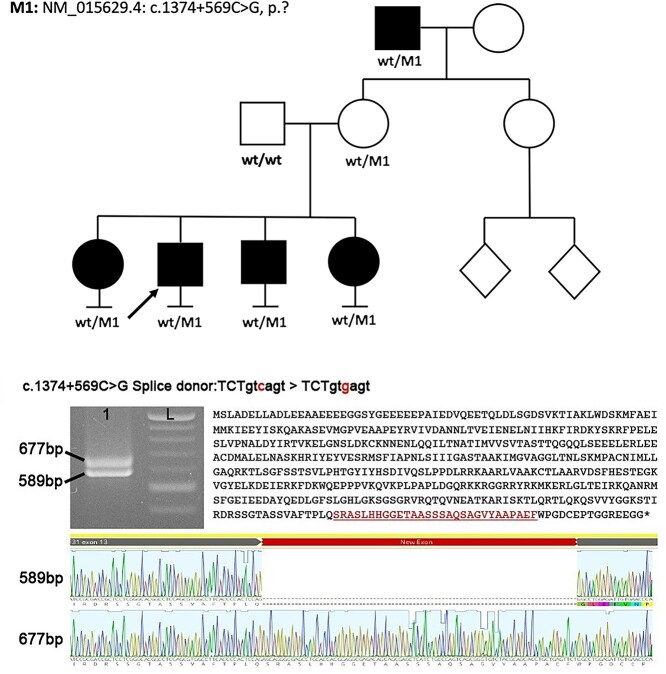
Analysis of mis-splicing owing to PRPF31 c.1374 + 569C > G. Pedigree of patient 1 showing a dominant family history with incomplete penetrance. Reverse transcription polymerase chain reaction (RT-PCR) and direct sequencing of patient 1 PRPF31 transcript (exon 8–14) derived from PAXgene RNA. Agarose gel analysis showing two distinct bands from RT-PCR amplification in patient sample. L: ladder. Direct sequencing of amplicons shows inclusion of 88 bp pseudoexon leading to a frameshift and premature termination.

Following negative coding variant analysis through the clinical pipeline and MDT discussion, *PRPF31* was deemed the most likely gene to harbor a pathogenic variant. A single rare variant (MAF < 0.001) was found in intron 10 of the *PRPF31* gene: (GRCh38) chr19:54129939C > G NM_015629.4: c.1374 + 569C > G. This variant was absent from the gnomAD dataset and predicted to cause a C > G change at the +3 position of a deep intronic donor site, creating a new splice donor site (nnsplice score 0.61: TCTgtcagt > TCTgtgagt). Further analysis of the upstream 500 bp revealed only weak splice acceptor sites with the closest being 88 bp upstream of the new splice donor site (nnsplice score 0.37; Fig. 2).

Reverse transcription polymerase chain reaction (RT-PCR) of the *PRPF31* transcript using oligonucleotide primers spanning exon 8–14 resulted in two distinct amplicons from whole blood RNA of patient 1, compared with a control sample. Direct sequencing of the two amplicons showed a wild-type spliced transcript and a larger fragment incorporating a deep intronic cryptic exon of 88 bp, matching the by *in silico* predicted pseudoexon. The inclusion of 88 bp into the transcript after exon 10 would lead to a reading-frame shift and a premature termination codon: p.(Gly459Serfs^*^46), indicating that this is likely to represent a loss of function (LOF) allele. This variant was also found in the patient’s three affected siblings, carrier mother and affected maternal grandfather.

One additional candidate variant (c.-9 + 1G > A) was identified in *PRPF31* in an individual with adRCD (Patient 2, GC14595, Tables 1 and 2) and his similarly affected mother. This variant is absent from the gnomAD dataset and located at a canonical splice donor site in the 5′ untranslated region (UTR). It was not possible to obtain a fresh sample from the patient for further analysis.

Patient 3 (GC 21538) reported decreased vision since his late 20s and was diagnosed with cone-rod dystrophy (CRD). His acuity slowly decreased over a follow-up period of 17 years, and rod-derived symptoms such as nyctalopia and peripheral field loss started to arise during his 30s. He did not have extra-ocular manifestations that would suggest a syndromic disease at the age of 45 years.

**Figure 3:**
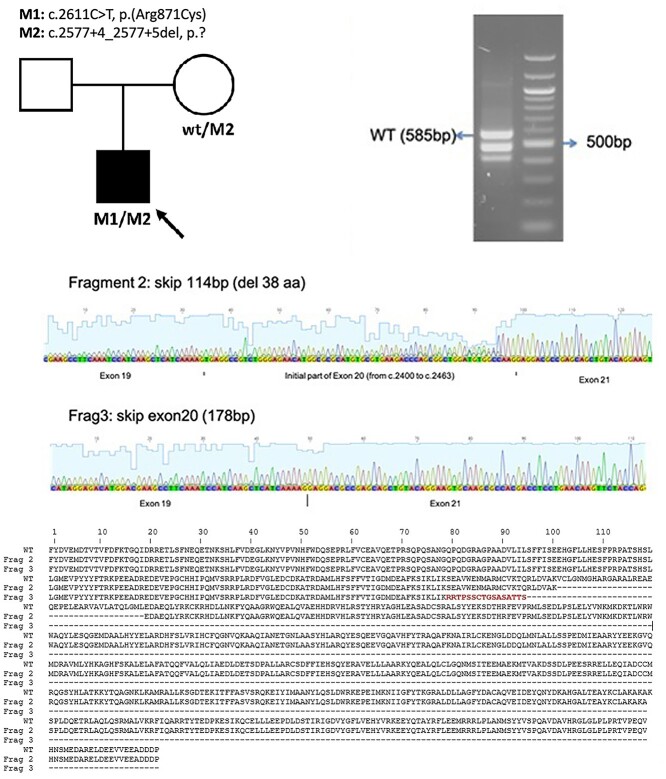
Analysis of mis-splicing owing to IFT140 c.2577 + 4_2577 + 5del. Pedigree of patient 3. Nested reverse transcription polymerase chain reaction (RT-PCR) showing multiple fragments amplified from patient 3 cDNA derived from PAXgene RNA sample. Bands [lane 3 (patient) fragment 1 (wild-type, WT), 2 and 3, L: ladder] were purified and sequenced by direct Sanger sequencing. Fragments 2 and 3 showed partial skipping of exon 20 and complete skipping of exon 20, respectively, with clustal alignment of the fragments shown.

Retinal dystrophy panel genetic testing identified a heterozygous previously reported missense variant in *IFT140*, c.2611C > T, p.(Arg871Cys). This is present in 4/253206 alleles in gnomAD v2.1 and reported three times in ClinVar (two pathogenic, one variant of uncertain significance, VUS). Non-coding region analysis revealed the intronic variant c.2577 + 4_2577 + 5del, predicted to abolish the canonical splice donor site of intron 20 AAGgtgag > AAGgtggg (nnsplice score: 0.00). This variant was also found in the unaffected patient’s mother. Nested RT-PCR of the patient’s sample showed multiple fragments amplified: wild-type, partial skipping of exon 20 and complete skipping of exon 20 (Fig. 3).

Patient 4 (GC 18850) was reviewed shortly after a full-term birth owing to horizontal nystagmus and poor fixation. At the age of 6 months, he was noticed to have bilateral retinal folds with subretinal fluid accumulating around the optic disk (Fig. 1B). His retinal phenotype and poor visual acuity (VA; 1.6 LogMAR in the right eye and no light perception in the left eye) were stable throughout the years. His maternal grandfather had a history of childhood exudative vitreoretinopathy and bilateral retinal detachment in his 20s, implying an X-linked inheritance pattern. Taken together, the clinical findings were suggestive of Norrie disease or X-linked familial exudative retinopathy, secondary to a damaging variant in *NDP*. His case remained unsolved following coding variant analysis as part of the NIHR-RD study. Rare variant analysis of the entire *NDP* gene region revealed a single variant, chrX:43817961C > T: NM_000266.4: c.-70G > A. It was absent from the gnomAD dataset, unique to the proband within the NIHR-RD (approx.13000 alleles) and absent from the 100KGP dataset. This variant, although outside the promoter region, is located in a transcription factor-binding region and DNase hypersensitive region spanning the UTR of exon 2 (UCSC ChIP-seq TFB clusters track [strongest binding: CTCF, SMC3, RAD21] and UCSC DNase ChIP-seq metadata tracks [116/125 cell types], ENCODE datasets). However, we were unable to demonstrate any effect of the variant on the transcriptional activity of a luciferase reporter gene in HEK293 cells (data not shown). Subsequently, SpliceAI prediction of the variant effect showed a donor gain (score 0.40) GAGgtgaa > GAGgtaaa at position c.-72, which may introduce a splice donor site upstream of the start codon and therefore disrupt the correct transcript. RNA samples were unavailable to examine this.

**Figure 4:**
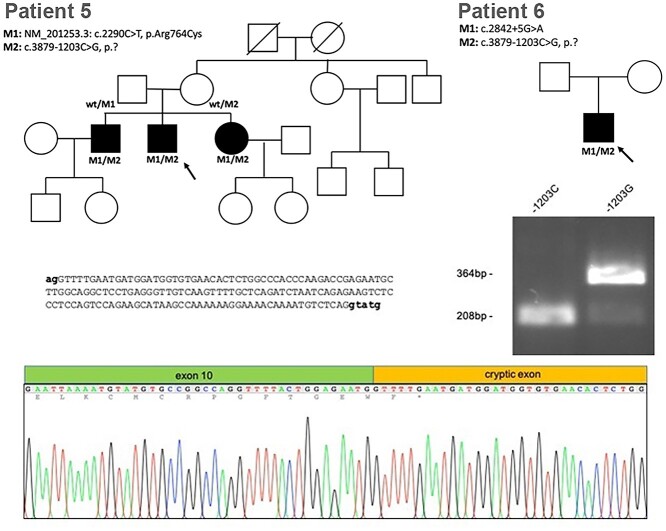
Minigene analysis of CRB1 c.3879-1203C > G. Pedigrees of patients 5 and 6. HEK293 cells transfected with CRB1-wt or CRB1-1203C > G containing minigene constructs. The PCR analysis shows splicing in of a 156-bp cryptic exon included in the CRB1-1203C > G transfected cells not present in the wild-type cells.

Patients 5 (GC 17009) and 6 (GC 3671) are two unrelated males, born to unaffected non-consanguineous parents, with visual disturbances since infancy and diagnosed with early onset severe retinal dystrophy (EOSRD). There was no family history of eye disease. Each had a retinal phenotype highly suggestive of *CRB1*-retinopathy (Fig. 1C). Patient 5 was recruited to the 100KGP along with his unaffected parents, and patient 6 was recruited to the NIHR-RD study as a singleton. Coding variant analysis (including splice regions) of all IRD genes identified only a single heterozygous pathogenic or likely pathogenic variant in *CRB1* in each case (patient 5: NM_201253.3: c.2290C > T, p.(Arg764Cys), patient 6: c.2842 + 5G > A). Non-coding region analysis of the *CRB1*-locus revealed an identical single candidate second variant in both cases (c.3879-1203C > G). This variant was predicted to create a deep intronic splice donor site (+1 position C > G, nnsplice score 0.96: CAGctatg > CAGgtatg). Analysis of the single-nucleotide variants (SNVs) co-inherited with the variant of interest showed that the haplotype was different in each case, demonstrating that the variant was likely to have arisen independently. Minigene analysis using a fragment of *CRB1* exon 10–11, including the 3.49-kb intron in a splicing minigene vector construct, and introducing the c.3879-1203C > G variant by site-directed mutagenesis demonstrated that a cryptic exon of 156 bp was included into transcripts when transfected into HEK293 cells (Fig. 4). This would be expected to lead to the frameshift consequence, p.(Cys1294Phefs^*^2). The near complete loss of normal splicing demonstrated by the minigene assay may suggest that this represents an LOF allele. An additional patient was found to harbor the same deep intronic *CRB1* variant in *trans* with an LOF variant (chr1:197427726A > T NM_201253.3*:* c.2401A > T p.Lys801^*^) through data sharing as part of the European Retinal Dystrophy Consortium (Patient 10, Supplementary Material, Table S1). Her symptoms started at the age of 2.5 years and, owing to nyctalopia and field constriction, she was diagnosed with EOSRD at the age of 4 years. On examination, she had para-arteriolar preservation of the RPE, pigmented bone spicules, attenuated vessels and generalized atrophy. Optical coherence tomography was typical of *CRB1-*retinopathy, with de-laminated inner retinal layers, retinal thickening, and loss of outer layers.

**Figure 5:**
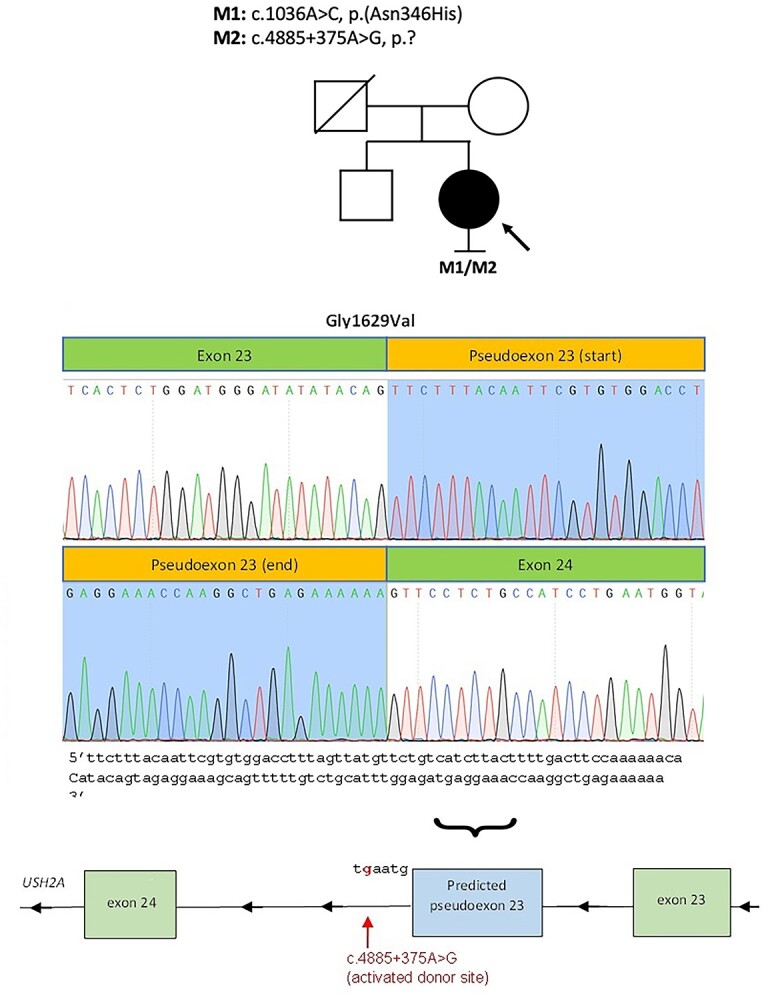
Cryptic exon inclusion consequent upon *USH2A* c.4885 + 375A > G. Prediction of pseudoexon inclusion owing to a strengthened deep intronic splice donor site (*USH2A* is on the reverse strand). Direct Sanger sequencing of PCR amplicons showed an alternate splice product comprising the inclusion of the predicted pseudoexon (130 bp), frameshift and premature stop codon (p.Gly1629ValfsTer52).

Patient 7 (GC 3769) was diagnosed with Usher syndrome type II, with hearing loss identified in early childhood and RCD diagnosed in her teenage years. The patient presented with a mild RCD phenotype, retaining a visual field of around 25 degrees at the age of 41 years, with preserved VA of 0.2 logMAR in the right and left eyes. Her fundus examination showed classic triad of RCD: pigmented peripheral bone spicules, vessel thinning and pale optic disks (Fig. 1D). OCT imaging revealed oedema and loss of the EZ line nasal and temporal to the fovea. Routine genetic testing identified the single heterozygous pathogenic variant in *USH2A*, chr1:216325412 T > G, c.1036A > C, p.(Asn346His) (NM_206933.4). She was recruited to the 100KGP with her unaffected mother and brother. Phasing of the missense variant in the unaffected family members demonstrated that the missing variant should have been inherited from her mother. Non-coding variant analysis of the *USH2A* locus focused on the maternal allele revealed 6 variants rare in the gnomAD dataset (MAF < 0.01), with only one, Chr1:216261980 T > C, c.4885 + 375A > G, surviving additional filtering (MAF < 0.001, manual curation for low-complexity region variants), and having a strong prediction for splice site effect, which strengthens a deep intronic splice donor site at the +5 position: AAAgtaaa > AAAgtaag). Direct Sanger sequencing of PCR amplicons spanning exons 22–24 of *USH2A* from nasal epithelial cell brushings showed an alternate splice product comprising inclusion of a pseudoexon (130 bp), predicted to lead to a frameshift and premature stop codon, p.(Gly1629Valfs^*^52), consequent upon *USH2A* c.4885 + 375A > G (Fig. 5).

Patient 8 (GC 18582) had been diagnosed as an adult with CRD; she developed loss of central vision and photophobia in her 40s. She had bilateral macular and peripheral retinal atrophy that progressed during the follow-up (Fig. 1E). Her disease was confined to the retina with no syndromic features.

Singleton GS as part of the 100KGP revealed a single pathogenic variant in the coding region of the *BBS10* gene: GRCh38 chr12:76345866_76345867del, c.2119_2120delGT, p.Val707^*^ (NM_024685.4). To identify a potential pathogenic *trans-*allele, an interrogation of the non-coding regions of the *BBS10* gene was performed, revealing a variant in the upstream region, Chr12:76348438dupG c.-80dupC (NM_024685.4), absent from the gnomAD v2.1 dataset. Given the position of this variant in the 5′ UTR, we hypothesized a possible regulatory effect. Inspection of the *BBS10* promoter region at the Eukaryotic Promoter Database (EPD; https://epd.epfl.ch/EPDnew_database.php) indicated the presence of two probable regulatory elements. EPD#1 is located at c.-106_c.-58, and EPD#2 is located at c.-829_c.-782, with the c.-80dupC variant located within EPD#1.

**Figure 6:**
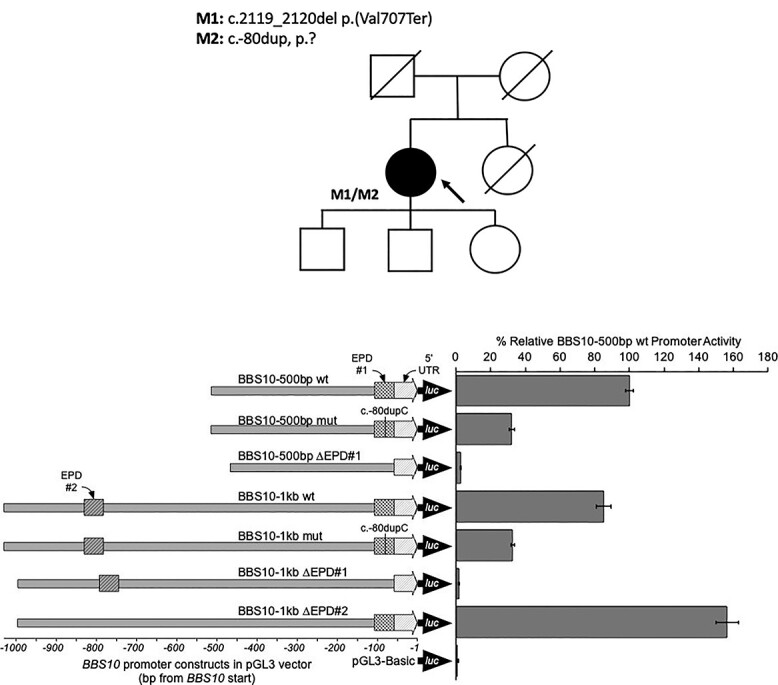
BBS10 promoter constructs and activity assayed by firefly luciferase expression in HEK293 cells. The various BBS10 promoter constructs are represented showing the relative positions of the c.-80dupC variant, 5′ UTR, and the EPD#1 and EPD#2 elements. The firefly luciferase reporter (luc) is also indicated. Expression levels are depicted relative to the wild-type BBS10 (500 bp) promoter with 95% confidence intervals indicated. BBS10 (500 bp) promoter variants are indicated by the fine crosshatching, while BBS10 (1 kb) promoter variants are indicated by bold crosshatching. Deletion of the EPD#1 promoter region resulted in complete loss of expression, whereas deletion of EPD#2 resulted in increased transcription (>1.5-fold). The mutant constructs showed an ~70% reduction in expression levels.

A series of *BBS10* promoter constructs driving firefly luciferase expression based on the pGL3 vector were made (Supplementary Material, Methods and Supplementary Material, Fig. S1). The BBS10 (500 bp) promoter construct contained only EPD#1, whereas BBS10 (1 kb) contained both EPD#1 and 2. When transfected into HEK293 cells, robust firefly luciferase expression was observed from both wild-type *BBS10* promoters—typically >5× that observed for the SV40-based pGL3-Control (data not shown). Relative to the BBS10 (500 bp) promoter, the BBS10 (1 kb) promoter displayed ~80% activity (Fig. 6). Introduction of the c.-80dupC variant led to an ~ 70% decrease in promoter activities, compared with their respective wild-type counterparts. This suggested that EPD#1 has a significant role in the expression of *BBS10* and that is affected by c.-80dupC. Analysis of other variants around the c.-80dupC position, especially c.-83 T > G, further supports the importance of this region as transcriptionally active (see Supplementary Material, Fig. S1). This was further confirmed by complete deletion of EPD#1 (∆EPD#1) in both the BBS10 (500 bp) and BBS10 (1 kb) constructs, with firefly luciferase levels falling to ~2.5% and ~1.5% of the respective wild-type (background activity of pGL3-Basic is ~0.6%). Interestingly, EPD#2 appears to have an inhibitory effect on the BBS10 promoter in this system, with the BBS10 (1 kb) ∆EPD#2 variant exhibiting firefly luciferase expression at nearly double that of the wild-type BBS10 (1 kb).

Oxford Nanopore Technologies (ONT) long-read sequencing of PCR amplicons spanning the entire genomic region (3 kb) enabled the phasing of the two variants to demonstrate they are in *trans*, in the absence of any family members for segregation analysis.

Patient 9 (GC 21400) was recruited for GS with both unaffected parents through the 100KGP. Clinically, he was noticed to have poor vision and nystagmus in his first year of life, which remained stable over his lifetime. He had good general health. His retinal exam showed a featureless fundus and macular atrophy (Fig. 1F). Full-field electroretinogram revealed poor rod and cone responses, and he was diagnosed with EOSRD. A single heterozygous missense c.2837C > A, p.(Ala946Glu) (chr17:8015395C > A, NM_000180.3) variant was identified in the *GUCY2D* gene. All other IRD genes had been excluded based on complete coding variant analysis. Non-coding variant analysis revealed a SNV upstream of the start codon of the *GUCY2D* gene (chr17:8002596 T > C, *GUCY2D* c.-148 T > C), shared with an unrelated affected individual recruited to the study independently (patient 11, see Supplementary Material, Data). Both individuals had a *trans-*allele harboring a candidate pathogenic variant in the gene (c.2837C > A, p.(Ala946Glu) and c.3043 + 5G > A, respectively). Therefore, the upstream variant was considered a good candidate in these unrelated cases. The analysis of transcription factor binding site motifs in the region (http://alggen.lsi.upc.es) showed four CRX binding sites in the vicinity of c.-148 (CBE#1–4, Supplementary Material, Data). The variant falls within the core 5′-TAAT’-3 sequence of CBE#1 (CTCTAATTA > CTCTGATTA) so we hypothesized that the variant may disrupt CRX binding to the *GUCY2D* regulatory region. Therefore, a series of *GUCY2D* upstream constructs were created comprising a 1-kb upstream region including the c.-148C > T variant and deletions of the CBE#1–4 (Supplementary Material, Data). Luciferase reporter assays in HEK293 cells required co-transfection with a *CRX* expression plasmid to induce expression from the *GUCY2D* promoter region, which demonstrated that the variant reduced expression by ~25% compared with wild-type and the effect was greater still in constructs with CBE#2–4 deleted with a reduction of ~40% expression with CBE#1 in isolation (see Supplementary Material, Methods and Supplementary Material, Fig. S2).

## Discussion

Application of NGS for investigation of Mendelian disorders is now widely accepted. When restricted to the coding regions of known genes, this fails to identify the complete causative genotype in many individuals. We report the likely disease-associated genotype of nine individuals who underwent GS and virtual gene-panel testing, followed by MDT-led targeted candidate gene non-coding variant analysis based on clinical and genetic data. In addition, two patients harboring recurrent variants in *CRB1* and *GUCY2D* were identified in other centers (patients 10 and 11).

Intronic mutations can alter canonical donor and acceptor splice sites and create cryptic splice sites, causing mis-splicing events such as exon skipping, pseudoexon inclusion or intron retention (30). Unless already proven pathogenic or found within canonical splice motifs at the intron/exon boundary, these variants may escape detection by standard sequencing approaches, requiring GS re-analysis and representing a diagnostic challenge (31). Nevertheless, owing to the high prevalence of IRD variant carriers (2), analyzing full genes to detect the second hit in IRD patients with monoallelic variants does not seem the optimal approach (32). We demonstrate here that narrowing the search by an MDT-prompted diagnostic awareness can help target the analysis to a small number of candidate genes and variants.

This study led not only to the finding of novel, intronic, damaging variants, but also to molecular diagnosis for the 11 patients studied here. We hypothesize that the spectrum of non-coding pathogenic variants may be somewhat limited. For example, for a pseudoexon to become active, the complete splicing architecture must be present, meaning that creating a deep intronic donor or acceptor site alone may have a limited effect in the absence of a partner site and thus the possibility of a single variant to accomplish this is indeed limited. A clue toward this from the current study is that the deep intronic variant in *CRB1,* c.3879-1203C > G, was found to be recurrent on independent genetic backgrounds. Previous findings in *ABCA4* suggest that pathogenic variants may occur at splice junctions of potential pseudoexons, detectable by RNA studies (22,33). Indeed, intronic pathogenic changes have been reported in many genes, with some recurrent or prevalent variants in *ABCA4*, *CEP290* and *USH2A* (22,24,34–37), further suggesting that the spectrum of intronic mutations is far more limited than that of coding mutations. Thus, it is possible that the intronic variants that are able to disrupt splicing are clustered at certain hotspots within the introns and many seemingly strong deep intronic splice sites may not lead to pseudoexon inclusion at all. Advanced splice prediction tools such as SpliceAI will prove useful to demonstrate this (38,39).

Proving that non-coding variants are damaging requires input from an expert MDT, supportive clinical data and functional studies. Undertaking such work is currently possible only through research projects and for high-priority cases, but should thereby provide enough evidence for the variant or region to be included in future diagnostic testing, ultimately leading to improved diagnostic yield for IRD. The relevance/prevalence of non-coding variants has been highlighted by Sangermano *et al*. and Khan *et al*., who found intronic *ABCA4* changes in 67 and 25% of the probands within their cohorts of unsolved Stargardt (STGD) and STGD-like individuals, respectively (26,40). *CEP290* c.2991 + 1655A > G is the most commonly found pathogenic variant in this gene, with 60–90% of the individuals with *CEP290*-related Leber Congenital Amaurosis (LCA) having it in at least one allele (active clinical trials are using CRISPR/Cas9—NCT03872479—and antisense oligonucleotide—NCT03913143—technologies to target this particular variant) (41). Similarly, *USH2A* c.7595-2144A > G was reported with a frequency of 4% among an Usher syndrome type 2A (USH2A)*-*patient cohort, representing the second most frequent *USH2A*-causing variant (37). Identifying and understanding these changes allows us to develop targeted therapies, which could correct aberrant transcripts (42).

Another potential site for pathogenic variants is the *cis*-acting regulatory sequence. The upstream region and 5′ UTR of any gene usually contains the promoter, enhancer, regulatory sites and transcription factor binding motifs, thus being important for gene regulation (43). Variants located in these regions have been found to be damaging in IRD genes such as *CHM, NMNAT1, EYS, LCA5, PRPF31, PRPF4* and *PCDH15* (23,25,28,29,44–46). In particular, *NMNAT1* has been found to harbor a hotspot for 5′ UTR variants (28). In this report, we have identified a damaging upstream variant in *BBS10* and an upstream variant in *GUCY2D* that disrupts binding of the transcription factor CRX. The *trans-BBS10* allele, p.Val707Ter, has previously been associated with classical BBS (47), and it is possible that the c.-80dupC allele is a hypomorph. As such, it would appear that ~15% of 2 N levels of wild-type BBS10 are sufficient to prevent manifestation of extraocular *BBS10*-related features. Similarly, reduced levels of wild-type GUCY2D expression may be related to the unusual presentation associated with the expected pathogenic p.Ala946Glu *trans-*allele (48). The 3′ UTR region, located downstream from the stop codon, serves as a binding site for microRNAs, which can also affect gene expression (27). Variants located in these regions were found to be damaging in Tourette’s syndrome (*SLITRK1*) (49) and Phosphoglycerate kinase 1 deficiency (*PGK1*) (50); however, these events are certainly rare and supporting evidence regarding their pathogenicity is hard to gather. Although such sites for pathogenic variants are likely to be even more limited than coding and cryptic splicing variants, these might still represent an important cause of disease.

Key to the analysis pipeline and discovery of candidate variants is the MDT role, which facilitates the interaction between the clinical team, genetic counselors, clinical scientists, and specialists in ophthalmic genetics. Interpretation of genomic data in isolation can miss vital clues for the elucidation of complex cases. An integrated clinical/genomic data analysis pipeline as a second stage following simple variant discovery/interpretation, including detailed medical history, retinal imaging and functional testing, in combination with targeted variant interrogation and input of experts in ophthalmology and genomics, can lead to a diagnostic uplift in cases that would otherwise remain unsolved (20,51). This synergy between clinicians and specialists in genetics was also noted to increase the diagnostic yield in other areas such as pulmonology (52), neurogenomics (53), nephrology (54), and prenatal diagnosis (55). Regular scheduled MDT meetings to discuss potential clinical diagnostic tests and analyze genetic results is undoubtedly of good practice and leads to increased quality of patient care, with faster, more efficient diagnoses, and contributing to new disease-causing gene discovery. Nonetheless, many molecular laboratories, in particular commercial and service laboratories, may present difficulties exploiting detailed clinical data, therefore missing out on critical information that can highlight gene targets and drive the analysis.

In conclusion, our work provides novel non-coding variants in several IRD genes, which will enrich retinal panels and hopefully contribute to decrease the missing genetic etiology in this field. It also underscores the immense benefit of MDT, decreasing diagnostic time and cost. Furthermore, we pose a question with regard to the diversity of viable intronic splice changes. Future worldwide collaborations will help determine if said hypothesis stands.

## Materials and Methods

This study adhered to the tenets of the Declaration of Helsinki and was approved by the Institutional Review Board and ethics committee of Moorfields Eye Hospital (REC 12/LO/0141). Informed consent was obtained from all participants prior to the inclusion in the study. For patients and relatives recruited for the 100KGP, informed consent for GS was obtained in accordance with approval from the HRA committee East of England-Cambridge south (REC 14/EE/1112). For patients recruited to the NIHR-RD study, participants provided a written informed consent and the study was approved by the East of England Cambridge South national institutional review board (13/EE/0325).

All patients underwent clinical variant interpretation using virtual gene panel analysis and MDT discussion. Patients were selected for study based on having a clinical presentation that was likely to correspond to a particular gene and, in the case of recessive diseases, a heterozygous pathogenic or likely pathogenic variant in a gene that was deemed highly likely to harbor the disease variant.

Subsequent non-coding variant analysis was performed on the gene of interest in all available family members’ GS data to establish phase, where possible. Rare variants (allele frequency ≤ 0.001) were identified and considered to be candidate pathogenic changes. Variants were further manually curated for quality (using the Integrative genome viewer, IGV (56)), low complexity, repetitive and polymorphic regions (using gnomAD). *In silico* analysis was performed on surviving variants including splice prediction (nnsplice, SpliceAI), conservation score and regulatory/promoter region prediction, depending on the variant position in the gene. Where a single variant was identified, no specific prediction threshold was applied for splice prediction analysis.

Compelling variants were functionally assayed using PAXgene stabilized whole blood for messenger RNA (mRNA) transcript analysis to identify mis-splicing in ubiquitously expressed genes (*PRPF31*, *IFT140*) or mRNA transcript analysis on total RNA extracted from nasal epithelial cells (*USH2A*), where expression is absent in whole blood. Minigene analysis was performed for retina-specific genes (*CRB1*) and luciferase reporter gene assays was undertaken for putative regulatory region variants (*BBS10, GUCY2D*). Methods can be found in the Supplementary Material, Data.

ONT single molecule sequencing was performed on PCR amplified gDNA to phase variants. Briefly, PCR amplification was performed with oligonucleotide primers spanning the *BBS10* gene and reactions were purified for ONT library preparation with AMPure XP magnetic beads (Beckman Coulter). Up to 50 ng of purified PCR product was used for library preparation using the SQK-LSK109 kit (Oxford Nanopore Technologies, Oxford, UK). Libraries were run on Flongle (Oxford Nanopore Technologies, Oxford, UK) flowcells for approximately 12 h. Reads were basecalled with Guppy v2 (Oxford Nanopore Technologies, Oxford, UK) and aligned to the human genome build GRCh38 using Minimap2 (https://github.com/lh3/minimap2). Samtools (https://samtools.github.io) was used to generate indexed sorted BAM files for visualization of individual read data using IGV.

Ophthalmic examination included visual acuity (using logMAR visual acuity charts), spectral domain optical coherence tomography (Spectralis, Heidelberg Engineering Ltd, Heidelberg, Germany), ultra-widefield (UWF) color fundus photography (200°, Optos plc, Dunfermline, UK) and fundus autofluorescence (FAF) imaging, performed with 55° Spectralis or UWF Optos. Four patients had visual electrophysiology testing, including full-field and pattern electroretinography, which incorporated the International Society for Clinical Electrophysiology of Vision (ISCEV) standards (57).

## Supplementary Material

Supplementary_data_final_ddac227

## Data Availability

Further details of the GS and ES data presented in the study are available via direct contact with the corresponding author. Data accessibility information for the 100KGP is available online (www.genomicsengland.co.uk/join-a-gecip-domain).
