# Supplementary material for: Multidisciplinary team directed analysis of whole genome sequencing reveals pathogenic non-coding variants in molecularly undiagnosed inherited retinal dystrophies
Source: Hum Mol Genet. 2022 Sep 9;32(4):595–607. doi: 10.1093/hmg/ddac227 (PMC9896476; doi:10.1093/hmg/ddac227)
Supplement: Supplementary_data_final_ddac227 [file supplementary_data_final_ddac227.docx]

**Supplementary methods**

- **mRNA analysis**

PAXgene RNA stabilised whole blood samples were collected from affected patients and stored at -70 degrees until extraction. RNA was purified using the standard protocol from the manufacturer (Qiagen, PAXgene blood RNA extraction kit). Total RNA was reverse transcribed using random hexamers, followed by PCR with oligonucleotide primers targeting a region of the relevant transcript flanking 1-3 exons up and downstream of the affected splice junction were used to amplify the transcript. Nested PCR primers were used where bands were faint or absent following a single round of PCR. Agarose gel analysis was performed to separate alternately spliced bands which were then purified and sequenced using the forward and reverse primers and standard protocols.

- ***PRPF31 c*.1374+569C>G**

Nested PCR was performed (exons 1-14 followed by 8-14) on an affected patient cDNA sample from PAXgene RNA stabilised blood. Sequencing of PCR amplicons was performed by following agarose gel purification of bands. Primers are detailed on Table S1.

- ***CRB1* c.3879-1203C>G**

A minigene consisting of CRB1 exons 10 to 11 including the intervening intron was cloned into pcDNA3 vector and the 1203C>G SNP was introduced into the wild-type intronic DNA sequence by site directed mutagenesis.

Plasmid DNA was extracted using ZymoPURE II Plasmid Midiprep Kit (Zymo Research). Wild-type and mutant CRB1 constructs were transfected into HEK293 cells with TransIT-LT1 (Mirus Bio) according to the manufacturer’s instructions. 24 hours after transfection, RNA was extracted and transcribed into cDNA.

- ***USH2A* c.4885+375A>G**

Patient nasal epithelial cells were collected using a cytology brush inserted into the inferior turbinate of each nostril. Cells were washed in Hank’s Balanced Salt solution, pelleted and RNA extracted using the RNeasy mini kit (Qiagen). RT-PCR was performed as described above.

- **Promoter plasmids and general luciferase assays**

Promoter constructs were designed to enable directional cloning into the promoter-less firefly luciferase reporter vector pGL3-Basic (Promega). Gene-of-Interest (GoI) promoters were amplified from human genomic DNA using Q5 Hot Start High-Fidelity 2X Master Mix (New England Biolabs), using primers that had an *Xho*I site appended to the forward primer(s), and a *Hind*III site appended to the reverse primer - indicate by underlined lowercase. PCR products were resolved by 1.5 % agarose gel electrophoresis, and the amplicons excised and gel purified (Monarch DNA Gel Extraction Kit, New England Biolabs), prior to cloning into pSC-B-amp/kan (StrataClone Blunt PCR Cloning Kit, Agilent). Mutations were introduced by site directed mutagenesis (SDM) (Q5 Site-Directed Mutagenesis Kit, New England Biolabs). Promoters were then sub-cloned into *Xho*I-*Hind*III sites of pGL3-Basic to become pGL3-GoI variants. Sequence fidelity was verified at all stages by Sanger sequencing (Source BioScience).

Plasmid DNA was prepared using the ZymoPURE II Plasmid Midiprep Kit (Zymo Research). HEK293 cells (maintained in high glucose DMEM supplemented with 10 % foetal bovine serum, 100 units/ml penicillin and 100 µg/ml streptomycin), were plated in 96-well plates at a density of 25,000 cells/well in 100 µl of media. After 18 hours, the cells were transfected with 100 ng of plasmid DNA /well using TransIT-LT1 (Mirus Bio) according to the manufacturer’s instructions. Briefly, per well, 50 ng of pRL-CMV (Promega) was complexed with 50 ng of either pGL3-Control or pGL3-Basic (both Promega), or the various pGL3-GoI promoter constructs, with TransIT-LT1 in Opti-MEM (Gibco), prior to dilution with maintenance media to 100 µl. The complete transfection media was then used to replace the overnight maintenance media in the 96-well plates. Transfections proceeded for 24 hours prior to luciferase analysis. Luciferase levels were assayed using the Dual-Glo Luciferase Assay System (Promega), and measured using a microplate luminometer (Berthold), with a 5 second integration per well. Six firefly luciferase (pGL3 variant) transfections per condition per plate were normalised to their respective *Renilla* luciferase level and averaged. These 96-well plate transfections were repeated a minimum of 3 times.

- ***BBS10* c.-80dupC**

*BBS10* promoter region fragments of 508bp (BBS10-500bp) and 1027bp (BBS10-1kb) were PCR amplified using the primers mentioned in Table S1. The c.-80dupC variant was introduced by SDM and the deletion of the EPD#1 and EPD#2 regulatory elements was achieved in a similar manner. Prior to normalisation, a x1.1 correction factor was applied to the BBS10-1kb constructs to compensate for decreased copy number relative to the BBS10-500bp constructs in the 50ng transfection. Similarly, a x0.91 correction factor was applied to the pGL3-Basic vector. Additional variants identified in gnomAD v3.1.2 (<https://gnomad.broadinstitute.org/>) around the c.-80dupC in EPD#1were also introduced by SDM. See Figure S1.

- ***GUCY2D* c.-148T>C**

A 1014 bp GUCY2D promoter fragment was PCR amplified using primers mentioned in Table S1. The c.-148T>C variant was introduced by SDM and deletions of the CRX binding elements (CBEs) was achieved in a similar manner. Plasmid DNA complexes/well consisted of 5 ng of pRL-CMV, 47.5 ng of pCMVk-CRX (a CRX expression plasmid), and 47.5 ng of the various pGL3-GUCY2D constructs. Prior to normalisation, to compensate for decreased copy number relative to the GUCY2D constructs, a x0.83 correction factor was applied to the pGL3-Basic vector. See Figure S2.

|  | | Forward primer | | Reverse primer |  |
| --- | --- | --- | --- | --- | --- |
| *PRPF31* | PCR: 1830bp  PRPF31_1F: GACCAATCAGAGAGTAGCTTTGC  PCR: 589bp  PRPF31_8_RT_F: 5’ CCACACCGGCTACATCTACC 3’ | | PCR: 1830bp  PRPF31_14R: AAAGACAAGGCATGATGAAGACC  PCR: 589bp  PRPF31_14_RT_R: 5’ TGGGTTCACAATCTCCAGGC 3’ | | |
| *IFT140* | Nested PCR:  1st PCR: 995bp  IFT140_18F: GATCCTGTCCTTCTTCATTTCCG  2nd PCR: 585bp  IFT140RT_F: TGCCACCACATCCCTCAGAT | | Nested PCR:  1st PCR: 995bp  IFT140_24R: CCTCCTGGCTCTCGTACTGG  2nd PCR: 585bp  IFT140RT_R: GTCCTCCGACAGCATCCTG | | |
| *CRB1* | CRB1SDMFor 5’-AATGTCTCAGgTATGCTTGGTATC-3’  Cryptic splicing in intron 10:  CRB1FOR 5’-GCAGATTACCCTCAACAGT-3’ | | CRB1SDMRev 5’-TTGTTTTCCTTTTTTGGCTTATG-3’  Cryptic splicing in intron 10:  CRB1REV 5’-GCAAAGGCAACATCACAGAGGC-3’ | | |
| *USH2A* | Nested PCR primers:  PCR: 474bp  USH2A_21F: GGGTATTGTAAATTTCCCAGCTC  PCR:243bp  USH2A_22F: TTTGCACTTCAGTTGAAGAAGG | | Nested PCR primers:  PCR: 474bp  USH2A_25R: AGGTTCCCAAATAGCTGACGG  PCR:243bp  USH2A_24R: CCCTCCCAGAAAGACTCCTG | | |
| *BBS10* | BBS10-500bp:  BBS10_500_Pro_X1F, 5’-ttctcgagGTGGACAGGAACATGCGTGAATCC-3’  BBS10-1kb:  BBS10_1kb_Pro_X1F, 5’-ttctcgagAGCATACTGTTGATCACCGACGTG-3’  c.-80dupC introduction:  BBS10_SDM_F, 5’-cCGTTTCCGGCCGTTCCCA-3’  Deletion of the EPD#1 and EPD#2:  BBS10_delEPD1_F, 5’-GTTTTCGGTCGGCCCGGG-3’  BBS10_delEPD2_F, 5’-CGGAGGTTGCAGTCAGCC-3’  Additional *BBS10* EPD#1 variants:  BBS10-66CtoT_SDM_F, 5’-TCCGGCCGTTtCCACCCCTGT-3’  BBS10-71CtoT_SDM_F, 5’-CCGTTTCCGGtCGTTCCCACC-3’  BBS10-83TtoG_SDM_F, 5’-TCCCCGCCTAgTCCGTTTCCG-3’  BBS10-84AtoC_SDM_F, 5’-CTCCCCGCCTcTTCCGTTTCC-3’ | | BBS10-500bp and BBS10-1kb:  BBS10_Pro_H3R, 5’-aaaagcttATCTGGGCCGCTTCCCCTTTTTGACC-3’  c.-80dupC introduction:  BBS10_SDM_R, 5’-GAATAGGCGGGGAGAAAACCC-3’  Deletion of the EPD#1 and EPD#2:  BBS10_delEPD1_R, 5’-CCAGGAGTCGAAGCCGGG-3’  BBS10_delEPD2_R, 5’-ATTACAGGCATACGCCACTG-3’  Additional *BBS10* EPD#1 variants:  BBS10-66CtoT_SDM_R, 5’-AACGGAATAGGCGGGGAGAAAACC-3’  BBS10-71CtoT_SDM_R, 5’-AATAGGCGGGGAGAAAACCC-3’  BBS10-83TtoG_SDM_R, 5’-GAAAACCCGGAAGCCAGGAG-3’  BBS10-84AtoC_SDM_R, 5’-AAAACCCGGAAGCCAGGA-3’ | | |
| *GUCY2D* | *GUCY2D* 1Kb promoter:  GUCY2D_5UTR_X1_F, 5’-ttctcgagAATCAGCAAGAGGGACTTGGAACC-3’  c.-148T>C introduction:  GUCY2D_Pro_SDM_F, 5’-CTTGGGGAGAcTAAGGGCTCT-3’  CRX binding element deletion:  CBE#1_SDM_F, 5’-CTTGGGGAGAcggAGGGCTCTGG-3’  CBE#2_SDM_F, 5’-TTAAGGACCCccgTCAGCTTGGGGAGATTAAGGG-3’  CBE#3&4_SDM_F, 5’-CCTCCCTACCccgcggAGGACCCTAATCAGC-3’  GUCY2D_CBE234_SDMF, 5’-gacccccgTCAGCTTGGGGAGATTAAGGGCTCTG-3’  c.-148T>C mutation into CBE#1 of the *GUCY2D* deleted CBE#2,3&4:  GUCY2D_CBE234T-C_F, 5’-CTTGGGGAGAcTAAGGGCTCTGG-3’ | | *GUCY2D* 1Kb promoter:  GUCY2D_5UTR_H3_R, 5’-aaaagcttTGCCGGCTTCTGCGAACACAGACG-3’  c.-148T>C introduction:  GUCY2D_Pro_SDM_R, 5’-CTGATTAGGGTCCTTAATTAGGTAG-3’  CRX binding element deletion:  CBE#1_SDM_R, 5’-CTGATTAGGGTCCTTAATTAG-3’  CBE#2_SDM_R, 5’-TTAGGTAGGGAGGGGCGG-3’  CBE#3&4_SDM_R, 5’-GGCGGGCTAGTGGAGGAT-3’  GUCY2D_CBE234_SDMR, 5-ctccgcggGGTAGGGAGGGGCGGGCT-3’  c.-148T>C mutation into CBE#1 of the *GUCY2D* deleted CBE#2,3&4:  GUCY2D_CBE234T-C_R,5’- CTGACGGGGGTCCTCCGC-3’ | | |

**Table S1**: Primers utilised in the experiments mentioned throughout the manuscript.

**Figure S1**: Additional variants identified in gnomAD v3.1.2 around the *BBS10* c.-80dupC variant. **(A)** Allele information. **(B)** Variant locations in the EPD#1 (uppercase) relative to the start codon. (C) Luciferase activity of the variants relative to the wild-type 500bp BBS10 promoter in the pGL3 vector backbone in HEK293 cells (± 95% C.I.).

**Figure S2**: *GUCY2D* promoter analysis. **(A)** Location of the potential CRX binding elements (CBEs; highlighted, core 5’-TAAT-3’ underlined) relative to the *GUCY2D* transcription start site (TSS). The position of the c.-148T>C variant is indicated in CBE#1. EPD sequence is italicized. Consequences of the c.-148T>C variant on the core and CBE deletions are indicated. CBE deletion was achieved by the substitutions 5’-TAAT-3’ > 5’-CCGT-3’ following Pittler *et al*. (2004, Journal of Biological Chemistry, 279, 19800-19807). **(B)** Luciferase activity of the *GUCY2D* promoter variants relative to the wild-type promoter in the pGL3 vector backbone in HEK293 cells (± 95% C.I.). **(C)** Summary table of decreases in observed *GUCY2D* promoter variant activity.
